# Changes in Iron Metabolism Induced by Anti-Interleukin-6 Receptor Monoclonal Antibody are Associated with an Increased Risk of Infection

**DOI:** 10.3390/ph12030100

**Published:** 2019-06-28

**Authors:** Renata Ribeiro, Frederico Batista, Filipe Seguro Paula, José Delgado Alves

**Affiliations:** 1 Immune Response and Vascular Disease—CEDOC, Chronic Diseases Research Center, NOVA Medical School, 1150-082 Lisbon, Portugal; 2 Systemic Immune-mediated Diseases Unit (UDIMS), Department of Medicine IV, Hospital Professor Doutor Fernando Fonseca, 2720-276 Amadora, Portugal

**Keywords:** iron, hepcidin, ferroportin, Interleukin-6, infection, rheumatoid arthritis

## Abstract

(1) Background: Treatment of patients with rheumatoid arthritis (RA) with an anti-IL-6 receptor (anti-IL-6R) monoclonal antibody (tocilizumab) has been found to influence iron metabolism. The objective of the present study was to ascertain whether changes in iron metabolism induced by anti-IL-6R biologic therapy were independently associated with an increased infection risk. (2) Methods: A prospective longitudinal study of patients with RA treated with tocilizumab was conducted. RA patients treated with an antitumor necrosis factor α monoclonal antibody were also included as a control group. The primary outcome was occurrence of infection during the first 24 months of biologic therapy. (3) Results: A total of 15 patients were included, with a mean age of 51.0 ± 4,1 and 73.3% (n = 11) female. A multivariate survival regression model, adjusted for confounding factors, was fitted for each of the iron metabolism variables. Hazard ratios for being above the median of each parameter was considered. Transferrin saturation above the median value (>32.1%) was associated with a higher infection risk (HR 4.3; 95%CI 1.0–19.69; p = 0.05). Similarly, although non-significantly, higher serum iron was strongly associated with infection occurrence. (4) Conclusions: This study identified a probable association between infection risk and higher serum iron and transferrin saturation in patients with RA on anti-IL-6R biologic therapy. We suggest that both these parameters should be considered relevant contributing factors for infection occurrence in patients on anti-IL-6R therapy.

## 1. Introduction

Infection is a frequent complication during the natural clinical evolution of rheumatoid arthritis (RA), contributing to disease-associated morbidity and mortality [1]. Age-adjusted mortality in RA might be increased about twofold when compared to the general population, and infectious diseases are one of the leading causes of death [2]. This infection risk has been found to be influenced by several disease-related conditions, patient-associated comorbidities, and the immunomodulatory drugs used. Scores of disease activity, such as the disease activity score 28 (DAS-28), have been found to correlate with infection risk [1,3]. Corticosteroids, synthetic disease-modifying anti-rheumatic drugs (DMARDs), and biologic DMARDs may also suppress the immune response, which might translate into an additional risk for infection [2,4]. 

In the complex pathophysiology of RA, interleukin-6 (IL-6) overproduction is of major relevance. IL-6 is a pleiotropic cytokine with a role in eliciting the acute-phase response in the liver, B-cell proliferation and antibody production, and T-cell differentiation and cytotoxicity [5]. Chronic inflammatory states, such as the one present in RA, through increased levels of IL-6 and other cytokines, result in an increased transcription of the HAMP gene in the liver, which encodes hepcidin [6]. After binding to its receptor in hepatocytes, IL-6 induces a Janus kinase 1 (JAK1)-mediated phosphorylation of STAT3, which in turn activates the transcription of the HAMP gene [6]. Hepcidin is an iron regulatory hormone [7] whose best-documented activity is binding to and promoting endocytosis and degradation of ferroportin, the only known iron exporter expressed in cells involved in iron metabolism [7,8,9]. High concentrations of circulating hepcidin, characteristic of inflammatory states, results in low expression of ferroportin in duodenal enterocytes, hepatocytes, Kupffer cells, and splenic macrophages, decreasing iron export to the extracellular fluid and plasma [7,8]. The result, called hypoferremia of inflammation, has been proposed to be evolutionarily advantageous [9]. Three adaptive functions have been proposed for the hypoferremia of inflammation: inhibition of extracellular bacterial growth by sequestering available iron (considering that most infectious agents need to scavenge iron from the host to multiply); protection from the toxicity caused by high levels of iron and heme released during tissue necrosis and erythrocyte destruction; and increased transferrin capacity to bind to iron released during infection and inflammation [6,8].

Considering the IL-6 overproduction observed in RA, one of the biologic DMARDs used successfully for its treatment is tocilizumab, a humanized anti-IL-6 receptor (anti-IL-6R) monoclonal antibody designed to block IL-6 signaling [10]. In patients with RA treated with tocilizumab, infection is considered the most frequent adverse event and respiratory infections are the most frequently documented [11,12,13]. Although clinical trials initially reported low infection rates associated with tocilizumab therapy in patients with RA [11,12,13], some observational studies of real-life registries have found higher infection rates [5]. Comparisons of infection rates between anti-IL-6R and antitumor necrosis factor α (TNFα) therapy have also been conducted, with some observational cohort studies finding a higher infection rate in tocilizumab [14,15].

The IL-6 signal blockade may suppress hypoferremia of inflammation [5]. It has been demonstrated that blocking IL-6 or TNFα pathways with tocilizumab or anti-TNFα monoclonal antibodies resulted in significantly lower serum hepcidin and significantly higher serum iron [16]. However, the effect on hepcidin was noted to be stronger in patients treated with tocilizumab when compared with patients treated with anti-TNFα monoclonal antibodies [16]. Analysis of the relationship between these parameters and infection risk observed in this type of patients has never been evaluated before.

The objective of the present study was to ascertain whether changes in iron metabolism induced by anti-IL-6R biologic therapy were associated with an increased risk of infection.

## 2. Results

This was a prospective longitudinal study of patients with a diagnosis of RA and treated with an anti-IL-6R biologic. RA patients treated with an anti-TNFα were also included as a control group. Both groups had a 24-month follow-up, with serum samples collected every semester and any infections during this period registered by clinical interview.

A total of 15 patients were included, with a mean age of 51.0 ± 4.1 and 73.3% (n = 11) female. All patients completed the follow-up time without censoring. Mean disease duration was 10.8 ± 9.5 years. Regarding biologic DMARD therapy, 10 patients were treated with an anti-IL-6R monoclonal antibody (tocilizumab) and 5 with an anti-TNFα monoclonal antibody (either infliximab, golimumab, or adalimumab). Baseline characteristics and additional drugs used at any time during follow-up are summarized in Table 1 and Table 2, respectively. Corticosteroid daily dosages were relatively low (median = 5 mg/day, IQR 5–10 mg/day, maximum of 20 mg/day, in prednisolone-equivalent dosages).

### 2.1. Iron Metabolism

**Figure 1:**
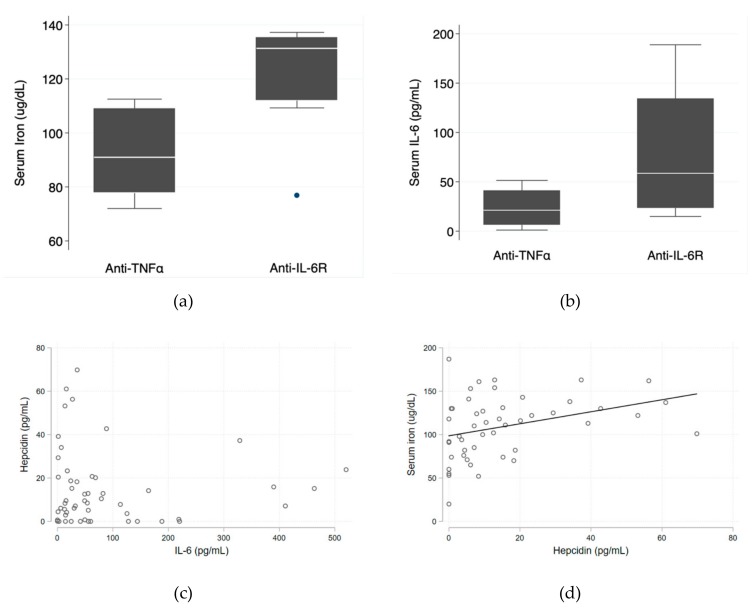
Iron metabolism. (**a**) Box plots showing median levels of serum iron in the two groups; (**b**) box plots showing median levels of serum IL-6 in the two groups; (**c**) inverse relationship between hepcidin and IL-6 levels; (**d**) positive correlation between serum iron and hepcidin, with the linear regression line shown. Please see text for details.

Median serum iron levels were significantly higher in the anti-IL-6R group (131.3 ug/dL (IQR 112.0–135.3 ug/dL) vs. 91.0 ug/dL (IQR 77.8–109.0 ug/dL); p = 0.028). Median serum IL-6 levels were also higher in the anti-IL-6R group (58.6 pg/mL (IQR 23.5–134.4 pg/mL) vs. 21.2 pg/mL (IQR 6.4–41.0 pg/mL); p = 0.090)—Figure 1a,b—which is consistent with the known mechanism of action of tocilizumab and corroborates good patient adherence. The relationship between serum IL-6 and hepcidin levels tended to be inverse (Figure 1c) and there was a direct positive relationship between hepcidin and serum iron levels (*p* = 0.018)—Figure 1d.

### 2.2. Infection Risk

During follow-up, a total of 29 infections were registered, 89.66% (n = 26) in the anti-IL-6R group and 10.34% (n = 3) in the anti-TNFα group. The types of infections are summarized in Table 3. Definitive microbiological identification of the infectious agents was not extensively pursued. The type of infectious agent (bacterial, viral, or fungal) was mainly presumptive considering clinical, analytical, and epidemiological data. Three infections were considered serious, 1 in the anti-IL-6R group and 2 in the anti-TNFα group. All serious infections were pneumonias requiring inpatient intravenous therapy. None of the infections resulted in death.

**Figure 2:**
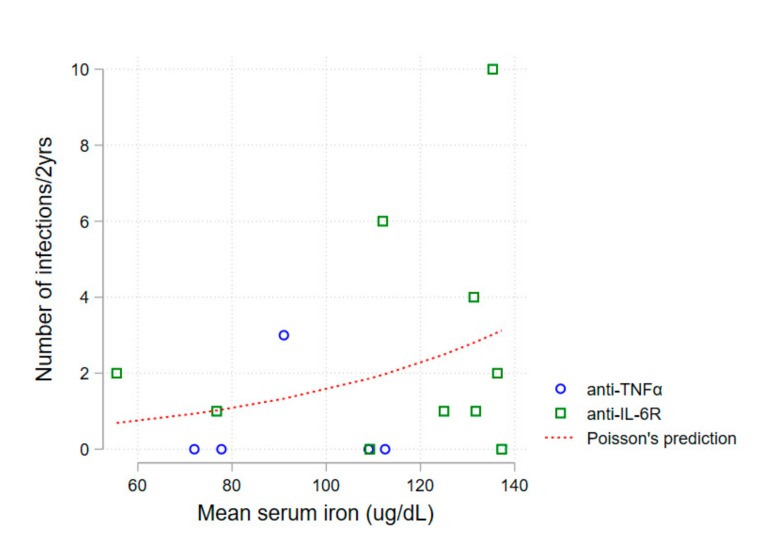
Relationship between mean serum iron level and number of infections during follow-up of patients both on anti-IL-6R (green squares) and anti-TNFα (blue circles) subgroups. The Poisson regression curve is depicted (dashed red), *p* = 0.030. Anti-IL-6R—anti-interleukin-6 receptor; anti-TNFα—antitumor necrosis factor α.

A first non-adjusted analysis of mean serum iron levels and number of infections during follow-up revealed an association between higher serum iron and a higher number of infections (Poisson regression, *p* = 0.030)—Figure 2.

Next, a multiple failure-time survival analysis was performed through fitting of an Anderson-Gill Cox proportional hazards regression model. Univariate analysis of patient’s characteristics identified a higher risk of infection in the presence of diabetes mellitus and a lower risk of infection in the presence of corticosteroid therapy—Table 4.

**Figure 3:**
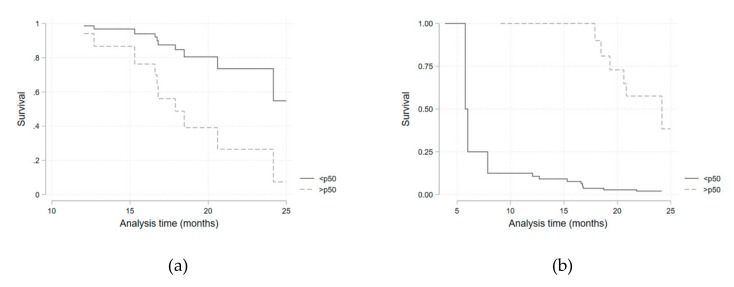
Kaplan–Meier survival curves for transferrin saturation (**a**) and ferritin (**b**) values above (solid lines) or below (dashed lines) the median.

**Figure 4:**
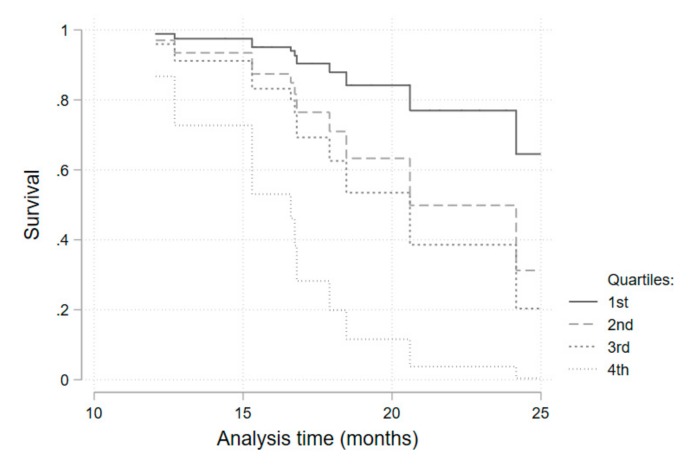
Kaplan–Meier survival curves for each quartile of serum iron values.

Considering the potential confounding variables identified in the univariate analysis, a multivariate survival regression model, adjusted for the presence of diabetes mellitus and for corticosteroid therapy, was fitted for each of the iron metabolism variables (Table 5, Figure 3). Hazard ratios for being above the median of each parameter distribution was considered. Transferrin saturation above the median value (>32.1%) was associated with a higher infection risk (HR 4.3; 95%CI 1.0–19.69; *p* = 0.05). This result was similar when a sub-analysis was performed in the anti-IL-6R group (HR 6.46; 95%CI 1.05–39.72; *p* = 0.044). Ferritin above the median value (>72 mg/dL) presented as a protective factor (HR 0.16; 95%CI 0.05–0.53; *p* = 0.003). As serum iron above the median value was, although non-significantly, still strongly associated with a higher infection risk (HR = 3.2 for all patients), we performed a quartile sub-analysis that showed a consistently higher risk of infection for each higher quartile (Table 6, Figure 4).

## 3. Discussion

This was a prospective longitudinal pilot study designed to evaluate whether changes in iron metabolism induced by anti-IL-6R biologic therapy were independently associated with an increased risk of infection in patients with RA. Higher infection risk was associated with higher serum iron and transferrin saturation.

Baseline characteristics were as expected for a cohort of patients with RA. Gender, median age, disease duration, and co-morbidities of patients on anti-IL-6R therapy did not differ when compared with a subgroup of patients on anti-TNFα. Non-biologic DMARD therapy use was also similar, with the exception of a significantly higher sulfasalazine use in the anti-TNFα group. Posterior analysis did not find a higher relative risk of infection in sulfasalazine users, and this difference was not considered relevant.

As expected, patients on anti-IL-6R monoclonal antibody therapy tended to have higher levels of free serum IL-6 than patients on anti-TNFα. These data are in accordance with drug pharmacodynamics and result from a diminished serum clearance of receptor-bound IL-6. The relationship between serum IL-6 and hepcidin levels seemed to be inverse, most probably as a result of a reduced IL-6 receptor-JAK1-STAT3 signal for HAMP gene transcription due to the IL-6-receptor blockade. Therefore, higher IL-6 serum levels (and consequently, a higher IL-6 receptor blockade) were associated with a lower hepcidin concentration. This was expected to have resulted in less endocytosis and degradation of ferroportin in duodenal enterocytes, hepatocytes, Kupffer cells, and splenic macrophages, higher iron efflux and, consequently, higher serum iron levels in patients on anti-IL-6R therapy. However, an unexpected positive linear correlation between serum iron and hepcidin was obtained, and higher levels of hepcidin were associated with higher levels of serum iron. This relationship may have been affected by the presence of unmeasured confounding regulatory factors of hepcidin production or may have derived from the stimulatory effect that serum iron has on hepcidin transcription in hepatocytes, through a mechanism of extracellular iron sensing [6].

In the univariate analysis of patient and disease-related characteristics, diabetes mellitus was the only statistically significant risk predictor for infection. The relationship between diabetes and infection risk is well-known in the general population and is expected to be similar in patients on biologic DMARD therapy. Corticosteroid therapy, on the other hand, tended to constitute a protective independent factor for infection occurrence in this study. It is commonly accepted that corticosteroid use increases infection risk [2], but it should be noted that the median daily dose per patient in our study was 5 mg/day. Such low dosages may have an immunomodulatory rather than an immunosuppressive effect, such that controlling chronic inflammation may translate into a lower infection risk. In light of this, survival analysis was adjusted for these two variables.

The relationship found between higher serum iron levels and transferrin saturation, and a higher infection risk was initially proposed and expected. Infective microorganisms use available iron as a catalytic component of enzymes that mediate many redox reactions, making it crucial for energy production and proliferation [6]. Concentration of iron in plasma and extracellular fluid decreases dramatically within hours of infection or other inflammatory stimuli through a cytokine-driven increase in hepcidin concentration [8]. This adaptative function of hepcidin is of particular importance when most common extracellular infective microorganisms are considered [8]. For intracellular agents (e.g., *Legionella pneumophila* and *Mycobacterium tuberculosis*) the hepcidin–ferroportin axis may play an opposing role, as iron release from infected cells into plasma could deprive microorganisms of the iron they need to grow [8]. Even without a definitive microbiologic identification, the infections reported here have a very low probability of being caused by intracellular organisms, based on clinical and epidemiological data. Furthermore, assistant physicians considered the majority of infections as being caused by extracellular bacteria and treated them accordingly and successfully. Infections were, as stated in previously published work [15], mainly respiratory, and none were considered serious. Transferrin saturation presented as a significant predictive factor for infection occurrence when above 32.1% (median). Although not statistically significant, a very consistent and progressive increase in the hazard ratio for each quartile of serum iron levels (when compared with the first quartile) was obtained. The reduced number of patients included may have contributed to a loss of statistical power. Additionally, a statistically significant protective effect of ferritin on infection risk was obtained. Higher serum ferritin probably reflects higher cellular ferritin and iron storage as a consequence of ferroportin degradation and lower serum iron availability. 

This study has some limitations. Blood sampling was not performed before biologic therapy was initiated, which limited the evaluation of the dynamic evolution of iron-related parameters. A small number of patients were included, and the anti-TNFα control group was smaller than the anti-IL-6R group, which might have limited statistical power. However, to the best of our knowledge, this investigation is the first to evaluate the relationship between iron metabolism and infection risk in patients with RA on anti-IL-6R biologic therapy, and it has consistently demonstrated that a trend in iron metabolism for a higher extracellular iron availability predicts infection occurrence. Given its intrinsically potent immunosuppressor effect, we would not suggest that elevated iron availability is the only mechanism through which tocilizumab increases infection risk, but our results show that it should be considered as a relevant contributing factor. Studies with larger cohorts are needed to confirm these data.

## 4. Materials and Methods

### 4.1. Study Design and Patient Eligibility

We conducted an observational prospective longitudinal study of patients with seropositive RA treated at the Systemic Immune-Mediated Diseases Unit in Portugal, under biologic DMARD therapy with either anti-IL-6R or anti-TNFα. All patients gave written informed consent for participation in the study, which was conducted in accordance with the Declaration of Helsinki, and after approval by the Ethics Committee of Hospital Professor Doutor Fernando Fonseca. Patients entered the study at the start of biological therapy and were followed up for 24 months. All blood samples were collected in the morning without fasting. Patients were 18 years old or older. Exclusion criteria were pregnancy; chronic kidney disease KDIGO classification 4 or higher; chronic liver disease Child–Pugh B or higher; cancer; transfusion therapy, iron or erythropoietin supplementation during or three months before starting biologic therapy; primary or secondary immunodeficiency not related with the immunomodulatory therapy; refusal or inability to sign informed consent.

### 4.2. Assessments

We collected demographic, clinical, and therapy-related data such as comorbidities, disease activity, and type and time of exposure to additional non-biologic immunomodulatory therapy. Disease activity was measured with the DAS28 score [17] at the time of each blood sample collection.

Hepcidin and IL-6 plasma concentrations were measured using commercial ELISA kits (DY8307 and DY206, respectively—R&D systems, Minneapolis, MN, USA). All other biochemical and hematological parameters were measured using standard laboratory techniques. Blood samples from infection episodes were excluded.

### 4.3. Outcomes

The primary outcome was occurrence of infection during the first 24 months of biologic therapy. Infections were identified through clinical interviews during each regular follow-up visit at our clinic. Infections were grouped into respiratory (including upper and lower respiratory tract infections); genitourinary (including cystitis), pyelonephritis, and vulvovaginitis; skin and soft tissue (including cellulitis), erysipelas, abscesses, and wound infections; ear infections (including otitis media and externa); gastrointestinal infections (including acute gastroenteritis); and eye infections (such as purulent conjunctivitis). Serious infections were defined as requiring hospitalization or intravenous antibiotics.

### 4.4. Statistical Analysis

Differences in dichotomous variables between treatment groups were assessed by Spearman’s or Chi-square tests as appropriate. Continuous variables were compared using t-tests or Mann–Whitney tests as appropriate, judging normality by a Shapiro–Wilk test. As an initial analysis, the number of infection events was counted and directly compared between patients, as all patients had the same follow-up time (24 months), and the association with mean serum iron values was tested using a Poisson’s regression. Next, a survival analysis considering multiple failure-time data, with all patients starting follow-up at the start of biologic drug administration (time 0). No censoring events resulted in all patients being followed until the end of the 24 months. An Anderson–Gill Cox regression model was used to assess each independent covariate to the outcome of interest (infection rate) to check for potential confounders. Variables with a *p*-value of <0,1 were selected for inclusion in the multivariate model for each of the components of iron kinetics available. The association of each variable of interest (serum iron, transferrin, transferrin saturation, total iron binding capacity, ferritin, hepcidin, and IL-6) was performed by stratifying it to above/below the median. Kaplan–Meier survival curves were developed for significant associations. Sub-analyses with a quartile stratification was also possible. Analyses were conducted with the use of STATA software, version 14.0 (StataCorp).

## Figures and Tables

**Table 1:** Baseline characteristics.

Baseline Characteristics	All (n = 15)	Anti-IL-6R (n = 10)	Anti-TNFα (n = 5)	*p*-value
**Patient-related characteristics**				
Age (years) Female	51.0 ± 4.1	51.1 ± 5.7	51.0 ± 5.4	0.9986
	11 (73.3%)	7 (70%)	4 (80%)	0.6900
**Disease-related characteristics**				
Disease duration (years)	10.8 ± 9.5	10,7 ± 9.7	10.9 ± 10.8	0.7815
First biologic therapy	8 (53.3%)	4 (40%)	4 (80%)	0.1573
**Comorbidities**				
Hypertension	7 (46.7%)	5 (50%)	2 (40%)	0.7237
Diabetes mellitus	3 (20%)	2 (20%)	1 (20%)	1.0000
Structural lung disease	3 (20%)	2 (20%)	1 (20%)	1.0000
Urolithiasis	2 (13.3%)	2 (20%)	0	0.2994
Tympanic perforation	2 (13.3%)	2 (20%)	0	0.2994
Gastrectomy	1 (6.7%)	1 (10%)	0	0.2994

Anti-IL-6R—anti-interleukin-6 receptor; anti-TNFα—antitumor necrosis factor α.

**Table 2:** Additional drugs used. ^1^

Drugs	All (n = 15)	Anti-IL-6R (n = 10)	Anti-TNFα (n = 5)	*p*-value
Proton pump inhibitor	9 (60%)	6 (60%)	1 (60%)	1.0000
Corticosteroid	11 (73.3%)	8 (80%)	2 (60%)	0.4250
Methotrexate	11 (73.3%)	6 (60%)	5 (100%)	0.1106
Sulfasalazine	4 (26.7%)	1 (10%)	3 (60%)	0.0461

^1^ At any time during follow-up. Anti-IL-6R—anti-interleukin-6 receptor; anti-TNFα—antitumor necrosis factor α.

**Table 3:** Type of infection in subgroups.

Anti-IL-6R ^1^		Anti-TNFα ^2^	
Respiratory	9	Respiratory	3
Genitourinary	8	-	-
Skin and soft tissue	4	-	-
Ear	3	-	-
Gastrointestinal	1	-	-
Eye	1	-	-

^1^ 26 infections in 8 patients. ^2^ 3 infections in 1 patient. Anti-IL-6R—anti-interleukin-6 receptor; anti-TNFα—antitumor necrosis factor α.

**Table 4:** Univariate survival analysis results.

Patient and Disease-Associated Variables	Hazard Ratio	*p*-value	95% Confidence Interval
**Constant baseline variables**			
Age	1.004915	0.800	0.9675943–1.043676
Male gender	0.2343794	0.124	0.0369132–1.488188
Disease duration	0.9324102	0.300	0.8169411–1.0642
Anti-IL-6R therapy	2.66909	0.335	0.3622716–19.66492
First-line therapy	0.9597915	0.955	0.2281255–4.038127
Hypertension	2.357254	0.295	0.4742625–11.71639
Diabetes mellitus	4.870464	0.023	1.249398–18.98629
Structural lung disease	1.031864	0.964	0.2653836–4.012092
Urolithiasis	2.224302	0.417	0.3229469–15.31991
Proton pomp inhibitor therapy	0.9087983	0.896	0.2176265–3.7951
**Time-varying variables**			
DAS28	0.7218875	0.357	0.3610093–1.443513
Erythrocyte sedimentation rate (mm/1hr)	0.9997664	0.994	0.9405976–1.062657
Corticosteroids	0.2458265	0.073	0.0529271–1.141771
Prednisolone daily dose (mg)	1.100572	0.394	0.8829673–1.371804
Methotrexate	0.3458809	0.185	0.0719138–1.66357
Methotrexate weekly dose (mg)	0.9233559	0.159	0.8264177–1.031665
Sulfasalazine ^1^	0.3954318	0.353	0.0558712–2.798691

^1^ Sulfasalazine daily dose relative risk estimates were not calculated due to the low number of patients on this drug (n = 4) and non-significant daily dose variation. DAS28—disease activity score 28.

**Table 5:** Multivariate survival analysis for iron metabolism parameters. Hazard ratios presented are for being above the median for each variable (relative to being below the median).

Iron Metabolism Parameters		Hazard Ratio	*p*-value	95% Confidence Interval
Serum iron	All	3.163589	0.089	0.8386898–11.93325
	Anti-IL-6R	2.570872	0.238	0.5356937–12.33799
Transferrin	All	1.314897	0.637	0.4223613–4.09354
	Anti-IL-6R	1.874245	0.447	0.3708102–9.4733
TIBC	All	1.314897	0.637	0.4223613–4.09354
	Anti-IL-6R	1.874245	0.447	0.3708102–9.4733
Transferrin Saturation	All	4.321854	0.050	0.999262–18.69222
	Anti-IL-6R	6.461889	0.044	1.051171–39.72334
Ferritin	All	0.600787	0.003	0.048141–0.5322946
	Anti-IL-6R	0.2356835	0.112	0.0395527–1.404372
Hepcidin	All	2.183736	0.465	0.2691971–1.71454
	Anti-IL-6R	9.408961	0.084	0.7420833–119.2973
IL-6	All	2.654745	0.271	0.4658854–15.12748
	Anti-IL-6R	2.173225	0.335	0.4478064–10.54677

TIBC—Total iron binding capacity; anti-IL-6R—anti-interleukin-6 receptor.

**Table 6:** Multivariate survival analysis for quartile distribution of serum iron.

		Hazard Ratio	*p*-value	95% Confidence Interval
All	Q1	(1)	-	-
	Q2	2.654559	0.363	0.3244244–21.72057
	Q3	3.633011	0.221	0.4601984–28.6806
	Q4	12.50757	0.085	0.7074825–221.1213
Anti-IL-6R	Q1	(1)	-	-
	Q2	0.1373109	0.239	0.0050287–3.749356
	Q3	0.6592579	0.596	0.1414934–3.071671
	Q4	1,112208	0,909	0,1806099–6,849054

Q—Quartile; anti-IL-6R—anti-interleukin-6 receptor.
